# Mixture Effects of Diesel Exhaust and Metal Oxide Nanoparticles in Human Lung A549 Cells

**DOI:** 10.3390/nano9091302

**Published:** 2019-09-11

**Authors:** Alessandra Zerboni, Rossella Bengalli, Giulia Baeri, Luisa Fiandra, Tiziano Catelani, Paride Mantecca

**Affiliations:** 1 POLARIS Research Center, Department of Earth and Environmental Sciences, University of Milano—Bicocca, Piazza della Scienza 1, 20126 Milan, Italy; rossella.bengalli@unimib.it (R.B.); g.baeri@campus.unimib.it (G.B.); luisa.fiandra@unimib.it (L.F.); 2 Microscopy facility, University of Milano-Bicocca, Piazza della Scienza 3, 20126 Milano, Italy; tiziano.catelani@unimib.it

**Keywords:** ultrafine particles, diesel exhaust, metal oxide nanoparticles, mixtures, cytotoxicity

## Abstract

Airborne ultrafine particles (UFP) mainly derive from combustion sources (e.g., diesel exhaust particles—DEP), abrasion sources (non-exhaust particles) or from the unintentional release of engineered nanoparticles (e.g., metal oxide nanoparticles—NPs), determining human exposure to UFP mixtures. The aim of the present study was to analyse the combined in vitro effects of DEP and metal oxide NPs (ZnO, CuO) on human lung A549 cells. The mixtures and the relative single NPs (DEP, ZnO, CuO) were characterized by transmission electron microscopy (TEM), dynamic light scattering (DLS) and inductively coupled plasma-optic emission spectroscopy (ICP-OES). Cells were exposed for different times (3–72 h) to mixtures of standard DEP at a subcytotoxic concentration and ZnO and CuO at increasing concentrations. At the end of the exposure, the cytotoxicity was assessed by 3-(4,5-Dimethylthiazol-2-yl)-2,5-Diphenyltetrazolium Bromide (MTT) and clonogenic tests, the pro-inflammatory potential was evaluated by interleukin-8 (IL-8) release and the cell morphology was investigated by fluorescence and transmission electron microscopy. The obtained results suggest that the presence of DEP may introduce new physico-chemical interactions able to increase the cytotoxicity of ZnO and to reduce that of CuO NPs.

## 1. Introduction

Several epidemiological studies show an increase in respiratory and cardiac morbidity and mortality due to the exposure to particulate matter (PM), especially related to pulmonary and cardiovascular diseases [1]. PM is a mixture of particles suspended in the air and is subdivided in PM_10_, PM_2.5_, and ultrafine particles (UFP) (<10 μm, <2.5 μm, and <0.1 μm, respectively) according to its “aerodynamic equivalent diameter”. Nowadays, UFP are the fraction of inhalable particles that largely impact on human health, since they can deeply penetrate in the respiratory system, reaching the profound lungs, where they interact with the alveolar epithelium, potentially cross the respiratory barrier and eventually reach secondary target organs [2]. In the recent years, increasing attention has been paid to the monitoring of UFP personal exposure [3], as well as to the experimental studies aimed at discovering the UFP toxicological impact.

Combustion processes represent the main sources of UFP, especially in urban areas. Among them, diesel exhaust particles (DEP), which are composed of a carbonaceous core and adsorbed organic compounds, including polycyclic aromatic hydrocarbons (PAHs), metals, and other trace elements [4], are the emissions that mainly contribute to urban PM. Another source of UFP are derived from non-exhaust sources, coming from tire and brake wearing [5] or from the unintentional release of engineered nanoparticles (NPs) in the environment. Since the exposure to DEP and to non-exhaust particles occurs simultaneously, it is inevitable to consider their possible interaction in biological systems. DEP toxicity has been studied both in vitro and in vivo and in 2012, the International Agency for Research on Cancer concluded that DEP is a carcinogen (Group I) [6]. Exposure to diesel exhaust is widespread all over the world and, in the last few years, the issue of health effects derived from DEP exposure was dealt with by national and international emission regulations for light duty vehicles emission. Non-exhaust particles derive from abrasive sources, which include tire and brake wear and abrasion of the road surface. Trace metals are an important source emitted by the above-mentioned processes in the urban environment [7]. The most abundant metals originating from brake wear are: Fe, Cu, Zn, Sn, and Sb [8], which mainly contribute to the non-exhaust fraction of PM. After Fe, Cu is the second element which is more representative of NPs emitted by brake wear [9], while zinc-based nanoparticles (Zn-NPs) mainly derive from tire wear [10,11]. Most of the metals are subject to corrosion either at room temperature or at high-temperatures and, by means of atmospheric oxidation, it is possible to produce passivating oxide layers on elemental metal Cu and Zn [12]. Metal oxides may have surface properties similar to their element counterparts [13]. Along with the extensive NPs industrial and medical application, especially of metal oxide nanoparticles (MeO), an accidental release in the environment of NPs could also occur during production and disposal operations. Nevertheless, even an unintentional exposure to these particles can occur, since they are present in everyday life goods, including textiles, food packaging, cosmetics, and paintings [14]. Finally, NPs have also been employed as engine fuel additives as an improvement in the abatement of greenhouse gases [15].

All of this evidence shows that, upon release and emission of nanomaterials (NMs) in the environment, these compounds may interact with other chemicals/contaminants, including metals and PAHs, that could lead to the occurrence of mixture effects and different toxicity on exposed organisms or in vitro systems. Furthermore, an initial classification of the possible NM–chemical interactions has been proposed by Naasz et al. [16], which also studied the transition of these interactions into specific biological effects.

In order to understand the possible mixture’s effects of DEP and non-exhaust particles we chose CuO NPs and ZnO NPs as representative MeO NPs. Studies showed that CuO NPs induce oxidative stress and cytotoxicity on airway epithelial cells [17,18], mostly through the Trojan horse mechanism and consequent cellular death or autophagy [19].

Other works demonstrate that ZnO NPs are toxic to different mammalian cells, thanks to their tendency to release in the cellular medium, free zinc ions, which exert their toxic effect in exposed cells [20].

Although several studies have been conducted on the toxicity of MeO NPs, the aspect regarding their potential interactions with other air pollutant UFP, in particular DEP, has still been poorly investigated. Many works have studied the toxicity of DEP. In vivo and in vitro studies report that DEP induced changes in markers of inflammation, including cytokines [21,22], oxidative stress and DNA damage [23]. Nevertheless, in our recent studies we demonstrated that different DEPs may induce different in vitro effects on the base of the chemical composition (PAHs and metals) [24].

The aim of the present study is to analyse the combined in vitro effects of a standard DEP (SRM^®^2975) and commercial metal oxides NPs (ZnO, CuO), on human lung cells A549 as a model of the respiratory system.

A549 cells derive from a human pulmonary adenocarcinoma and show properties such as surfactant production and transport-like AT-II cells in vivo, secrete cytokines, and perform phase I and phase II xenobiotic biotransformation similar to lung tissue [25,26]. For these reasons, they are the most representative cell line for lung tissue and are used for the screening of nanoparticles in inhalation toxicology. Furthermore, different regulatory frameworks for the safety evaluation of NMs, including the Organisation for Economic Co-operation and Development (OECD) Working Party on Manufactured Nanomaterials (WPMN) and the NANoReG project, A549 cells have been used as main the cell model common for several biological endpoints, making it a promising candidate cell model for regulation of in vitro testing of NPs [27,28].

Of relevant importance is to understand if and how ZnO NPs and CuO NPs may interact with DEP and how this may contribute in modifying the toxic effects. For this purpose, a set of tests was employed to evaluate dose-response cytotoxicity, colony forming efficiency (CFE), inflammatory response and morphology changes, and data were discussed in relation to the physicochemical properties of particles. More robust data regarding how single particles and mixtures elicit their effects may contribute to improving the hazard risk assessment of accidentally released UFP and engineered NMs.

## 2. Materials and Methods

### 2.1. Preparation of the Particle Suspensions

Commercial CuO (CAS 1317-38-0) and ZnO (CAS 1314-13-2) NPs, with size <50 nm, were purchased from Sigma-Aldrich (Sigma-Aldrich, Milan, Italy). Standard Diesel Exhaust Particles (DEP) were from the National Institute of Standard and Technology (NIST) (SRM^®^2975) (Sigma-Aldrich). Following preparation protocols already set up in our lab [19,29], NPs and DEP were weighed in a micro-balance (Sartorius, Goettingen, Germany) in sterile condition, under a laminar flow hood, suspended in sterile ultrapure water, and sonicated in an ultrasonic bath (SONICA Soltec, Milano, Italy) for 10 min.

DEP (2 mg/mL) was sonicated with a probe-type sonicator until it reached energy 3 kJ/s (Bandelin Sonopuls, Berlin, Germany), in order to obtain a well-dispersed suspension of particles. Suspensions were stored at room temperature, while DEP was stored at −20 °C for a period no longer than 15 days. The mixtures were freshly prepared before the experiments using a subcytotoxic concentration of DEP (100 µg/mL) and increasing concentrations of CuO and ZnO NPs (10, 15, 20, 25 µg/mL). Mixtures were sonicated in the ultrasonic bath (SONICA Soltec) for 10 min. The mixtures are indicated as DEP + CuO and DEP + ZnO and were produced by mixing DEP at 100 µg/mL with metal oxide NPs to achieve the final concentrations of CuO and ZnO used for the single NPs.

### 2.2. Physico-Chemical Characterization of the Particle Suspensions

#### 2.2.1. Transmission Electron Microscopy (TEM)

To study the particles’ morphology, primary size, and agglomeration state, a drop (5 µL) of the following particle suspensions was added in ultrapure water: DEP at 100 µg/mL, CuO and ZnO at 20 µg/mL, and mixtures of DEP (100 µg/mL) and CuO or ZnO (20 µg/mL) were pipetted onto Formvar^®^-coated 200 mesh copper grids. The excess of water was gently blotted by filter paper. Once dried, grids were observed under the TEM Jeol JEM-1220 (JEOL, Tokyo, Japan) operating at 80 kV, equipped with a charged-coupled device (CCD) camera.

#### 2.2.2. Dynamic Light Scattering (DLS)

The particles hydrodynamic size and surface charge were measured using a dynamic light scattering (DLS) (Malvern Zetasizer, Malvern, UK) technique, at a scattering angle of θ = 90°. ZnO, DEP + ZnO, CuO, and DEP + CuO mother suspensions were prepared using the method previously described, then diluted in Milli-Q water at a concentration of 100 μg/mL of DEP and 20 μg/mL of NPs.

#### 2.2.3. Inductively Coupled Plasma-Optic Emission Spectroscopy (ICP-OES)

The dissolution of the ZnO and CuO NPs in the cell medium was determined by quantifying the Zn^2+^ and Cu^2+^ in solution by inductively coupled plasma-optic emission spectroscopy (ICP-OES) after ultrafiltration of particle suspensions. Briefly, ZnO, CuO, and the mixtures DEP + CuO and DEP + ZnO were suspended in cell culture medium at a final metal oxide concentration of 20 μg/mL and incubated for 3 and 24 h at 37 °C. Then, before the analysis, the solutions were ultra-filtrated at 4000 g for 30 min, using centrifuge tubes, VIVASPIN, with a molecular weight cut-off of 10,000 Da (Sartorius StedimBiotech GmbH, Goettingen, Germany). The solution obtained from the ultrafiltration was then analysed by ICP-OEC (Optima 7000 DV; PerkinElmer, Waltham, MA, US).

### 2.3. Cell Culture and Treatments

Human alveolar epithelial cells, A549 (ATCC^®^ CCL-185, American Type Culture Collection, Manassas, VA, US), were grown at 37 °C, 5% CO_2_, and maintained in OptiMEM medium (Gibco, Life Technologies, Monza, Italy) supplemented with 10% heat-inactivated fetal bovine serum (FBS, Gibco) and 1% Penicillin/Streptomycin (Euroclone, Pero, Italy). For the experiments, cells (passages from 7 to 30), were seeded at a concentration of 1.6 × 10^4^ cells/cm^2^ on 6-well plates (Corning^®^) and grown up for 24 h. At the optimal confluence (80%), the culture medium was replaced with OptiMEM (1% FBS) and cells were exposed, directly adding particles suspension into the medium for different times of exposure (3, 24, 48, and 72 h) depending upon the different biological endpoints investigated. Experiments were assessed in at least three independent replicates.

### 2.4. Biological Responses: Cytotoxicity Assays

#### 2.4.1. Cell Viability: MTT Assay

For the 3-(4,5-Dimethylthiazol-2-yl)-2,5-Diphenyltetrazolium Bromide (MTT) assay, A549 cells were treated to DEP 100 µg/mL, CuO NPs, and ZnO NPs to 10, 15, 20 and 25 µg/mL, and a mixture of DEP 100 µg/mL + NPs (CuO or ZnO) at the concentration of 10, 15, 20 and 25 µg/mL. At the end of exposure at different time points (3 h, 24 h and 48 h), cells were rinsed with phosphate-buffered saline (PBS) and then MTT (Sigma Aldrich) solution, prepared in OptiMEM 10% FBS at a concentration of 0.3 mg/mL, was added to the cells and incubated for 3 h.

After the conversion of the substrate to a chromogenic product by metabolically active cells, the medium was removed and the purple MTT formazan crystals were solubilized with dimethyl sulfoxide (DMSO, Euroclone, Pero, Italy). The absorbance of each sample was measured with a multiplate reader spectrophotometer (Infinite 200Pro, TECAN, Männedorf, Switzerland) at 570 nm, using 690 nm as a reference wavelength. Cell viability, proportional to absorbance, was reported as relative decrease compared to the absorbance resulting from the control, considered as 100% of viable cells. Data are presented as mean ± standard error (SE) of at least three independent experiments.

#### 2.4.2. Colony Forming Efficiency Assay

The colony forming efficiency (CFE) assay is schematized in Supplementary Materials (Table S1). On Day 1, 200 A549 cells were seeded in 3 mL of OptiMEM 10% FBS in Petri dishes (Corning^®^, 60 mm diameter). According to Ponti el al. [30], after 24 h (Day 2), particle suspensions were added to the cells directly in the culture medium. Cells were treated with DEP 100 µg/mL, CuO NPs and ZnO NPs 10 and 20 µg/mL, and mixtures of DEP 100 µg/mL + NPs (CuO or ZnO) at concentrations of 10 and 20 µg/mL. Cells were treated for 24 h (from Day 2 to Day 3) and for 72 h (from Day 2 to Day 5). On Day 3 (24 h) and on Day 5 (72 h) the treatment was removed and replaced with fresh OptiMEM 10% FBS and cells were cultured for further 48 h (Day 8). At Day 8, the medium was removed and the colonies were fixed using a solution of 4% (*v/v*) formaldehyde in PBS, then stained using 0.4% (*v/v*) of Crystal Violet (Sigma Aldrich) 15% in aqueous ethanol for 5 min and washed several times with Milli-Q water. After drying, colonies were counted under a stereomicroscope, Carl Zeiss Stemi SV6 (Carl Zeiss SpA, Milano, Italy), and the percentage of the Colony Forming Efficiency (% CFE) was calculated as follows:

$$\%\mathrm{CFE}=\frac{\mathrm{average}\;\mathrm{of}\;\mathrm{number}\;\mathrm{of}\;\mathrm{colonies}\;\mathrm{in}\;\mathrm{treatment}}{\mathrm{average}\;\mathrm{of}\;\mathrm{number}\;\mathrm{of}\;\mathrm{colonies}\;\mathrm{in}\;\mathrm{the}\;\mathrm{control}}\times 100$$

In this work, cell colonies containing more than 20 cells were deemed adequate for the counting. Additionally, the area of the colonies was measured by AxioVision Real 4.8 software (Carl Zeiss Solutions, Jena, Germany) and expressed as µm^2^. To obtain this supplementary information on the colonies’ area, each plate was analysed by using the stereomicroscope. Experiments were performed in triplicate and parallel tests were performed in duplicate.

### 2.5. Quantification of Cytokine IL-8 Release

At the end of particles exposure (3 h and 24 h), cell culture media were collected and centrifuged at 10,000 rpm for 6 min to remove cell debris and particles suspended in the medium. The final supernatants were stored at −80 °C until analysis. IL-8 protein levels were determined according to the manufacturer’s instructions (IL-8, Life Technologies). The absorbance of each sample was measured by a multiplate reader (Infinite 200Pro, TECAN) at the wavelength of 450 nm and the amount of proteins in pg/mL was calculated based on standard curves. Data were expressed as pg/mL and experiments were performed in at least triplicate.

### 2.6. Morphological Analysis

#### 2.6.1. Fluorescence Microscopy

For the staining, A549 cells were fixed with 4% paraformaldehyde and, after washing with PBS, cytoskeleton actin was marked with rhodamine-phalloidin (1:150, Cytoskeleton Inc., Denver, CO, US). Nuclei were counter-stained with DAPI (4′,6-diamino-2-phenylindole, 1:100, Molecular Probes, Life Technologies). Finally, the slides were mounted with VECTASHIELD^®^ Antifade mounting medium (Vector Laboratories Inc., Burlingame, CA, US). The images were acquired with a reverse microscope (Carl Zeiss Axio Observer) and processed with the Zeiss ZEN (Blue edition) software.

#### 2.6.2. Transmission Electron Microscopy

For transmission electron microscopy analysis, cells cultured in 6-well plates and exposed to the different NPs as described above, were trypsinized, washed in PBS, and immediately fixed for 45 min in a 2% glutaraldehyde solution prepared in the same medium. Subsequently, cells were centrifuged for 10 min at 13,000 rpm in order to obtain a pellet, and fixative solution was replaced with 2% glutaraldehyde solution prepared in 0.1 M phosphate buffer (PB). After 1 h of fixation, cell pellets were washed with PB 0.1 M and post-fixed for 1 h in 1% Osmium Tetroxide solution prepared in PB 0.1 M. After several washes in PB 0.1 M, samples were transferred in Milli-Q water and then incubated overnight at 4 °C in 1% Uranyl Acetate aqueous solution. Finally, samples were dehydrated in ascendant with a series of alcohols, transferred in a final concentration of propylene oxide, and then embedded in Epon resin. After resin polymerization at 60 °C for 48 h, samples were cut with Rickert-Jung ultramicrotome and ultra-thin sections (70 nm) were collected on TEM grids. Samples were observed with a Jeol JEM 1220 Transmission Electron Microscope (JEOL, Japan), operating at 80 kV acceleration voltage and equipped with a Lheritier LH72WA-digital camera, and by a Zeiss SEM-FEG Gemini 500, operating at 30 kV in scanning transmission electron microscopy (STEM) mode (Zeiss, Germany).

### 2.7. Statistical Analysis

All the experiments were performed in independent triplicates, and data were reported as mean ± standard error (SE), if not otherwise specified. Statistical analyses were performed using One-Way ANOVA or unpaired *t*-test and relative post-hoc analysis with Sigma Stat 3.2. Values of *p* < 0.05 were considered statistically significant.

## 3. Results

### 3.1. Characterization of Nanoparticle (NP) Mixtures

**Figure 1:**
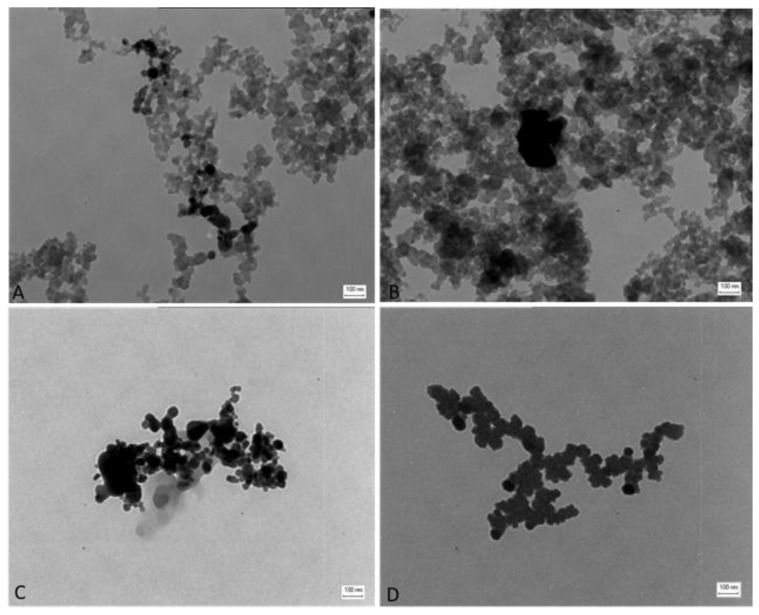
Morphological characterization of nanoparticles (NPs) and mixtures. Transmission electron microscopy (TEM) images of mixtures of: diesel exhaust particles (DEP) (100 µg/mL) and CuO (20 µg/mL) (**A**), DEP (100 µg/mL) and ZnO (20 µg/mL) (**B**), single NPs of: CuO NPs (**C**), ZnO NPs (**D**). Scale bars = 100 nm.

TEM analysis showed that the different particles suspended in solution form aggregates (Figure 1). The DEP sample showed a chain composition, typical of soot [24]. In a mixture with NPs, DEP forms particle aggregates with a chain structure too (Figure 1A,B). The single NPs of CuO (Figure 1C) and ZnO (Figure 1D) are also distinguishable in the mixtures DEP + CuO (Figure 1A) and DEP + ZnO (Figure 1B), since the NPs, being composed of heavy elements, are more electrodense compared to DEPs which are composed mainly of Carbon atoms. ZnO NPs showed an irregular shape and aggregates are composed of particles of similar size (Figure 1D), with jagged edges. Size distribution ranges from 10 to 40 nm with a mean diameter of 38 nm [31]. TEM analysis revealed that CuO NPs also have an irregular shape (Figure 1C), especially with spherical and rod-like particles. The size distribution ranges from 10 to 50 nm and mean diameter of 34 nm [19].

DLS analysis (Table 1) showed that the hydrodynamic diameter of all the particles increased when in culture medium compared to the data obtained in Milli-Q water, thanks to the tendency of NPs to form bigger aggregates in these media, due to the protein corona phenomenon. Nevertheless, the analysis in cell culture medium confirmed that the particles’ aggregates fall in the nanometric range. CuO NPs show a mean hydrodynamic diameter (z-average) higher than the other particles (464.67 ± 2 nm). DEP particles had the lower Polydispersity Index (PdI = 0.21 ± 0.01 in Opti-MEM) (Table 1), and therefore they were the more stable in the medium. Data showed that in culture cell medium, in the presence of DEP, the z-average is lowered for both CuO and ZnO NPs.

The data regarding ζ-Potential measurements in Milli-Q water (Table 1) showed positive potentials for ZnO and CuO NPs respectively, 25 ± 0.13 and 12 ± 0.6 mV, and negative potentials for their respective mixtures (−19 ± 0.21 mV for DEP + ZnO and −18 ± 0.09 for DEP + CuO). This behaviour could reflect the tendency of these NPs to have a poor stability in Milli-Q and to aggregate, since their surface charge does not allow a strong repulsion.

DEP instead have a negative ζ-potential (−35 ± 0.52 mV) (Table 1) that indicates the stability of this suspension.

ICP data (Table 2) show that the metal ions dissolution in cell medium is higher for ZnO NPs and for mixtures DEP + ZnO. In Table 2, the percentages of released ions are reported, and it is clear that dissolution was not complete for any of the particle suspensions. The release of Zn ions occurs with a higher percentage (69%) after 24 h of incubation with ZnO NPs, rather than the mixture of DEP + ZnO (62%). The release of Cu ions results are higher with the single NPs of CuO (43.3%) after 24 h of incubation, compared to the DEP + CuO mixture (26.7%). At 24 h, it is evident that the presence of DEP in both mixtures reduced the ions release.

### 3.2. Cytotoxic Effects

**Figure 2:**
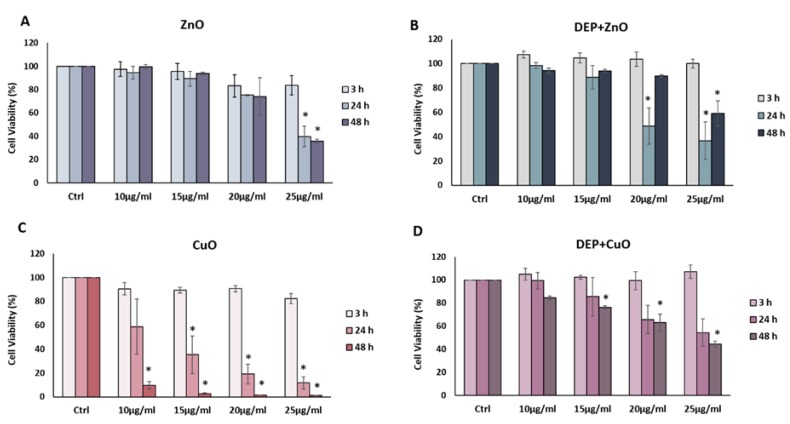
Cell viability: MTT assay. Histograms represent the percentage, with respect to control cells (Ctrl, 100%), of viable cells after the exposure to: 10, 15, 20, 25 μg/mL ZnO NPs (**A**), DEP 100 μg/mL + ZnO 10, 15, 20, 25 μg/mL (**B**), 10, 15, 20, 25 μg/mL CuO NPs (**C**), and DEP 100 μg/mL + CuO 10, 15, 20, 25 μg/mL (**D**). Data show the mean ± SE (n = 3). * Statistically significant with respect to the control according to One Way ANOVA; *p* < 0.05. Post hoc test: Tukey Test.

Results from the MTT assay performed at 3, 24 and 48 h after exposure to increasing concentrations (0, 10, 15, 20 and 25 µg/mL) of NPs and respective mixtures are reported in Figure 2. No significant decrease in cell viability was induced after 3 h of exposure to all the NPs tested, whereas a significant dose-dependent decrease of cell viability was observed after 24 h of exposure to CuO NPs at concentrations of 15, 20 and 25 µg/mL (Figure 2C). Interestingly, both ZnO and DEP + ZnO NPs at 24 h and 48 h induced a significant reduction of cells viability at the dose 25 µg/mL (Figure 2A,B). After 48 h of exposure, it is noteworthy that with the mixture, a partial recovery of cell viability was noticed. A strong cytotoxicity was observed after CuO NPs exposure (Figure 2C). At 24 h, a dose-dependent reduction in cell viability is evident, while at 48 h almost all cells resulted as not viable for CuO NPs doses ≥10 µg/mL. The mixtures of DEP + CuO also induced a significant decrease of viability with respect to the control, but at a lower extent compared to the effects induced by the single CuO NPs (Figure 2D). DEP exposure did not induce a significant viability reduction (Figure S1).

**Figure 3:**
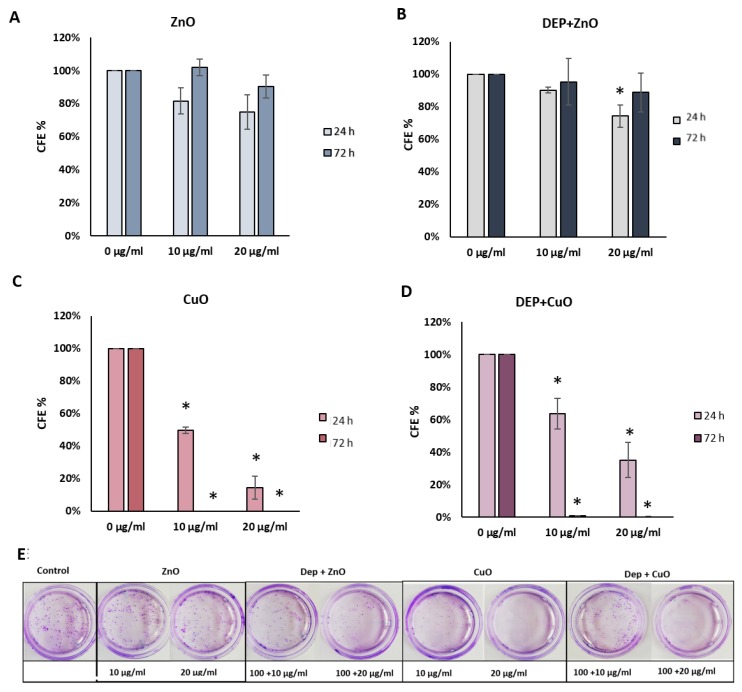
Colony forming efficiency (CFE) assay. Histograms represent the percentage of CFE calculated after the exposure for 24 h and 72 h to 10 and 20 μg/mL of ZnO (**A**), DEP 100 μg/mL + ZnO 10, 20 μg/mL (**B**), 10 and 20 μg/mL of CuO (**C**) and DEP 100 μg/mL + CuO 10, 20 μg/mL (**D**). Figure (**E**) shows the images of the Petri related to one experiment where cells were treated for 24 h. Data show the mean ± SE (n = 3). * Statistically significant with respect to the control according to One Way ANOVA; *p* < 0.05. Post hoc test: Tukey Test.

Figure 3 shows the percentage of CFE in treatment compared to control cells. After 24 h of exposure (Figure 3A), CFE confirmed the cytotoxicity of CuO NPs at the concentrations of 10 and 20 μg/mL, with a % CFE of about 50% and 14%, respectively. No colonies were detected after 72 h of exposure (Figure 3C).

A dose-dependent reduction of % CFE was also observed after exposure to DEP + CuO after 24 h (64% at 10 μg/mL; 35% at 20 μg/mL) and a higher reduction of colonies after 72 h, comparable to the effects of single CuO NPs. ZnO NPs induced a decrease of CFE, (82% at 10 μg/mL; 75% at 20 μg/mL) after 24 h, albeit not significant, while 20 μg/mL DEP + ZnO mixture induced a significant reduction of colonies at the same time point. After 72 h, ZnO NPs seemed to induce a recovery of colonies, in fact the % of CFE was higher compared to the results obtained after 24 h. The mixture DEP + ZnO induced a reduction of CFE (95% at 10 μg/mL, 89% at 20 μg/mL), slightly lower with respect to the single ZnO NPs. At 72 h, also with the mixture DEP + ZnO, a slight recovery was noticed. DEP induced 87% CFE after 24 h and 86% after 72 h of exposure (Figure S2).

### 3.3. Cytostatic Effects

**Figure 4:**
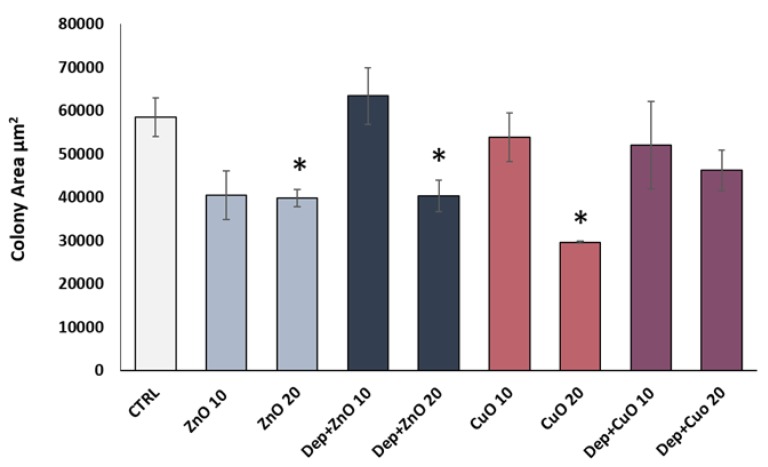
Cytostatic effect. Histograms represent the mean surface area (±SE) of A549 cell colonies treated for 24 h to ZnO NPs, DEP + ZnO mixture, CuO NPs and DEP + CuO mixture. * Statistically significant with respect to the control according to un-paired t test; *p* < 0.05.

The analysis of the mean surface area of the colonies was conducted in order to identify those treatments able to induce a cytostatic effect, which determines an inhibition of cell growth, and so a reduction in the colony area after 24 h of treatment. The results, expressed as the mean area of ten colonies counted, showed a decrease of the colony size after exposure to ZnO at the doses of 10 and 20 µg/mL and with DEP + ZnO at 20 µg/mL (Figure 4). Mixtures of DEP + CuO induced only a slight decrease in colony at both concentrations tested. The strongest cytostatic effect was observed for CuO NPs at 20 µg/mL. DEP alone did not induce a cytostatic effect compared to control cells (Figure S3).

### 3.4. Pro-Inflammatory Effects

**Figure 5:**
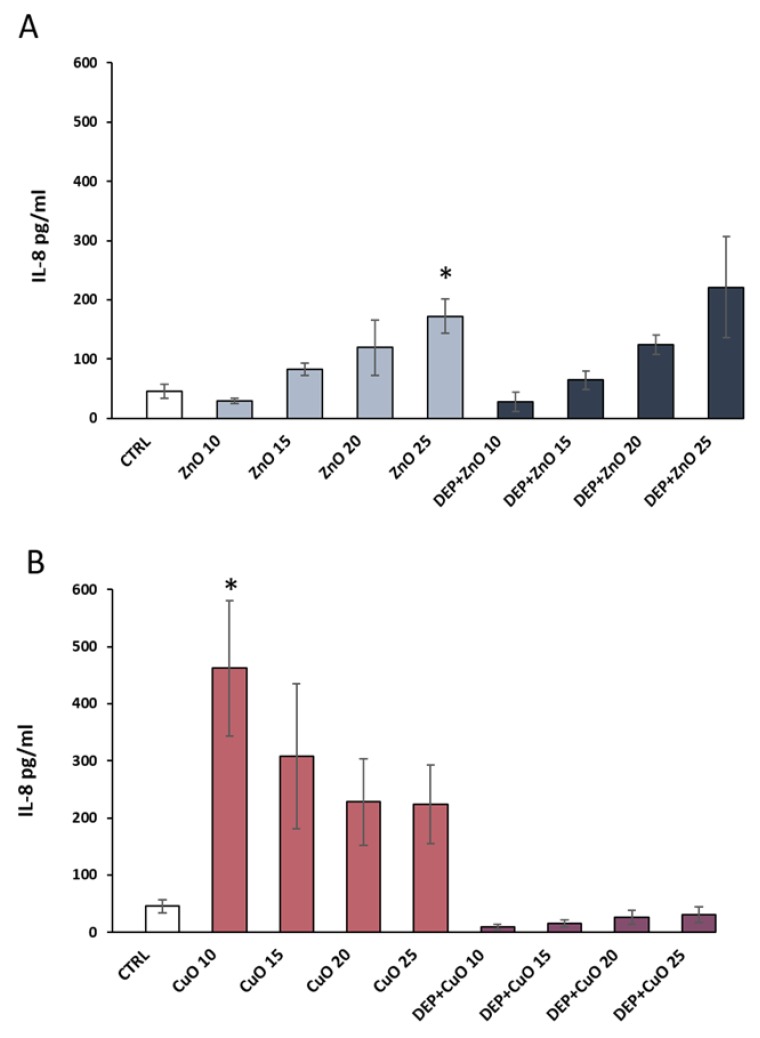
Inflammatory response. The release of the pro-inflammatory cytokine IL-8 was evaluated in A549 supernatants after the exposure for 24 h to 0, 10, 15, 20, and 25 µg/mL of ZnO (**A**) and CuO (**B**) NPs alone and mixed with DEP (100 µg/mL). Data are presented as pg/mL and the histograms represent the mean ± SE of at least three independent experiments. * Statistically significant according to the un-paired t test; *p* < 0.05.

The IL-8 release was investigated in order to evaluate the pro-inflammatory response related to NPs’ and mixtures’ exposure. ZnO and CuO NPs induced more IL-8 release compared to their respective mixtures with DEP. Data showed a significant increase in the release of IL-8 after exposure to ZnO NPs at 25 µg/mL (9.6-fold increase) (Figure 5A) and to CuO NPs at 10 µg/mL (15-fold increase) (Figure 5B) compared to control cells. DEP + ZnO mixture induced an increase in IL-8 release comparable to the one promoted by ZnO NPs, although no statistically significant values were reached. Interestingly, the CuO NP-induced inflammatory response seemed to be completely rescued by the presence of diesel particles (Figure 5B). DEP alone did not determine an increase in IL-8 release from exposed A549 cells (Figure S4). In addition, to confirm these results, we reported the pg/mL of IL-8 in relation to the total amount of proteins (mg) of each sample (Figure S5). Since the release of cytokines can occur at earlier time points, the amount of IL-8 secreted after 3 h of exposure to the different NPs and mixtures was assessed. A slight, though not significant, tendency of increased IL-8 release after treatment with ZnO and CuO (at the doses 20 and 25 µg/mL) at an earlier time point (3 h) was observed (Figure S6).

### 3.5. Cells Morphology

#### 3.5.1. Electron Microscopy

**Figure 6:**
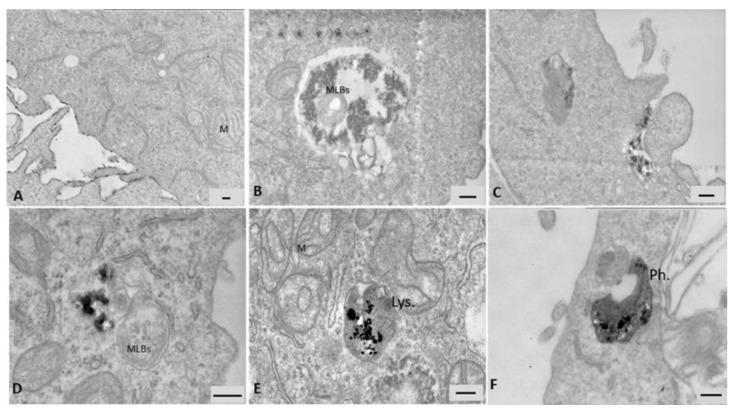
TEM analyses of A549 cells after 24 h of exposure to particles. (**A**) Ultrastructure of a control cell, scale bar = 150 nm. (**B**) ZnO (20 µg/mL) agglomerates internalized in large cytoplasmic vesicle, scale bar = 200 nm. (**C**) DEP (100 µg/mL) + ZnO (20 µg/mL) phagocytosis in cells, scale bar = 200 nm. (**D**) Internalization of DEP (100 µg/mL), scale bar = 200 nm, (**E**) CuO (20 µg/mL) agglomerates internalized in lysosome (Lys.), scale bar = 200 nm. (**F**) DEP (100 µg/mL) + CuO (20 µg/mL) internalized in a phagosome (Ph.), scale bar = 200 nm. M = mitochondria, MLBs = Multilamellar bodies.

Data from electron microscopy analysis showed the internalization of particles in A549 cells after 24 h of exposure (Figure 6). ZnO NPs were not taken up by cells very efficiently, only a few large cytoplasmic vesicles were observed containing cell debris and particulate material (Figure 6B and Figure S7). Although A549 are considered not-phagocytic cells, after the exposure to the DEP + ZnO mixture, phagocytosis processes consisting in protrusions of the cell membrane that engulf particles agglomerates have been detected (Figure 6C) and a large amount of particles was observed in several large endocytic vesicles and multivesicular bodies (Figure S7). The single DEP particles were efficiently internalized and ended up in cytoplasmic multivesicular bodies (Figure 6D). This suggests that in combination with DEP, the ZnO NPs uptake might have been improved. Figure 6E shows an agglomerate of CuO NPs in lysosomes, and the presence of swollen mitochondria (M) with loss of cristae is also noticeable. Cells exposed to the DEP + CuO mixture displayed particles internalized in a phagosome and were characterized by a similar altered ultrastructure (Figure 6F).

#### 3.5.2. Fluorescence Microscopy

**Figure 7:**
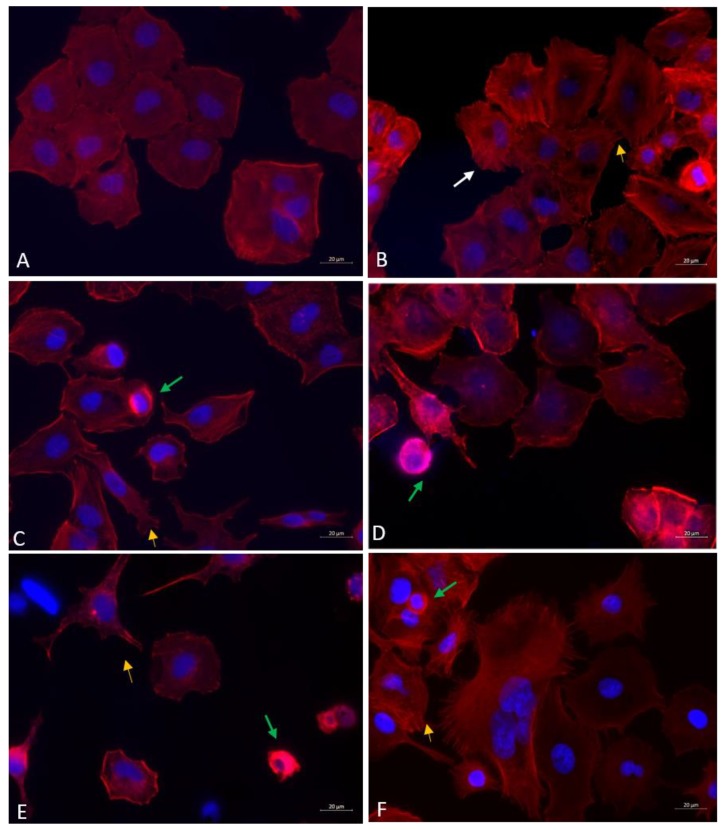
Fluorescence microscopy images of actin cytoskeleton. Control cells (**A**), DEP 100 µg/mL (**B**), ZnO 20 µg/mL (**C**), DEP + ZnO 20 µg/mL (**D**), CuO 20 µg/mL (**E**), DEP + CuO 20 µg/mL (**F**). Scale bar = 20 µm. White arrow: stress fibres, Orange arrow: microspikes (filopodia), Yellow arrow: lamellipodia, Green arrow: shrunk cells, Blue arrow: uropodia.

Fluorescence microscopy imaging of actin cytoskeleton shows that all the treatments induced a change in the regular shape of A549 cells, which have a uniform cobblestone appearance (Figure 7A). Protrusions of plasma membrane, such as lamellipodia and filopodia, are evident in cells exposed to DEP (Figure 7B), ZnO NPs (Figure 7C) and DEP + CuO mixture (Figure 7F). Stress fibres were evident in cells exposed to DEP (Figure 7B). Data also confirmed the CuO NPs cytotoxicity (Figure 7E), since a significantly lower number of viable cells with regular morphology was observed. Cells exposed to ZnO (Figure 7C) and CuO NPs (Figure 7E) appeared shrunken, as morphological evidence of cell death, and with uropodia as evidence of polarization and cells locomotion. Similar morphological alterations were appreciable in ZnO and DEP + ZnO-exposed cells, although the actin cytoskeleton of the cells exposed to the mixture appeared even more disorganized (Figure 7C,D). On the contrary, the cytoskeletal and nuclear morphology of the DEP + CuO-exposed cells resulted as more preserved with respect to the severely altered structures observed in the CuO NPs-exposed cells (Figure 7E,F).

## 4. Discussion

There is a great concern about the health risk associated with air pollution, especially to UFP. The issue concerning UFP emissions and the related health risk have been dealt with in the last year by regulating the circulation of the vehicle and by improving the technology of abatement systems for Diesel engine emissions. Europe has developed Euro standards which have continuously been lowered since 1993 with the Euro I to Euro VI, respectively [32].

Nevertheless, in addition to the exhaust source of pollution, there are also non-exhaust ones, which represent an important origin of particles in the nanometric range. Vehicles contribute to non- exhaust during the mechanical processes associated with driving, especially brake and tire wear. Furthermore, metal-based NPs are also used as fuel additives, such as cerium oxide and zinc oxide nanoparticles applied in order to mitigate the emission of particulates and greenhouse gases in the atmosphere [33].

Moreover, growing industrial activity based on nanotechnology represents an additional source of UFP that covers different classes of compounds, including MeO [34]. Since in the real conditions the population is exposed to a mixture of different particles, it is possible that the co-exposure to these mixtures can lead to a different toxicity compared to the well-known biological responses induced by the single compounds. Generally, there is a unanimous consensus on the necessity to demonstrate if the addition of NMs in the environment may alter the intrinsic toxicity of particles emitted in the exhaust [15]. A recent work showed that the co-exposure to benzo(a)pyrene (B(a)P) and silica nanoparticle (Si-NPs) synergistically potentiated the toxicological effects on endothelial cells, including DNA damage, oxidative stress, cell cycle arrest at the G2/M check point, and apoptosis [35].

In this perspective, the hazard assessment of NMs interactions with airborne UFP has been addressed here by a range of in vitro assays, aimed to evaluate the toxicity of two commonly used MeO NPs, CuO and ZnO, alone and in mixtures with standard diesel exhaust particulates derived from a light duty engine. DEP SRM^®^2975 was used at the concentration of 100 µg/mL (corresponding to 10 µg/cm^2^), which is a sub-cytotoxic dose typically used in in vitro experiments for assessing DEP effects in lung cells [36,37].

The morphological characterization of particles confirmed that they are in the nanometric range, with a tendency to agglomerate. DLS analysis showed that the hydrodynamic diameter for all particles increases in the culture medium. The CuO NPs formed the largest aggregates (465 nm), while the DEP + ZnO mixture formed the smallest aggregates with a hydrodynamic diameter of 207 nm. Data showed that in cell culture medium, the z-average is lowered for both CuO and ZnO NPs when in a mixture with DEP. We suppose that, in the cell culture media, the presence of DEP interferes with the NPs state of agglomeration, reducing it, with possible consequences on the interactions among NPs and cells. Since surface charge could be related to variation in their electrostatic interaction, we measured the ζ-potential of these particles. Obtained data showed that ZnO and CuO NPs have a charge of +25 mV and +12 mV respectively, while the values for DEP + ZnO and DEP + CuO mixtures are −19 mV and −18 mV, respectively. In both cases, the charge values were not sufficient to allow a repulsion between particles and data to justify the tendency of NPs to form aggregates. Several studies demonstrated that the dissolution of metal particles in the culture medium or in the biological environment/fluids resulted in the release of ions, which play a main role in their toxicity. Our results showed that the percentage of ions released by DEP + ZnO (62.5%) after 24 h of incubation in medium is similar to that of the single ZnO NPs (69.4%), unlike DEP + CuO, which instead showed a reduced percentage of released ions (26.7%) compared to the single CuO NPs (43.3%). While the release of Zn^2+^ ions from ZnO NPs seemed to not be influenced by the interaction with DEP, our data point out that diesel particles may somehow interact with CuO NPs, limiting the ions dissolution from their surface, thus reducing the bioavailability of free CuO NPs and Cu^2+^ ions. Of course, such speculation is worthy of additional physico-chemical studies.

The in vitro toxicity study has been performed on human lung A549 cells exposed to single particles or mixtures and the results have been interpreted considering the NP physico-chemical behaviour. The cytotoxicity was investigated using MTT, a standard metabolic assay based on a simple colorimetric reaction, but limited by the possible interference of absorbance with particles [38] and CFE assay. CFE has the advantage to be a label-free test, since it is a non-colorimetric and non-fluorescent assay, thus avoiding the possible interference with the particles. In addition, by using the CFE assay, it is possible to analyse particle-induced cytostatic effects that lead to the reduction in the colony area. Moreover, the CFE assay has been recognized as a reliable and standardised method for the in vitro toxicity assessment of NMs [30].

The combined results from the MTT and CFE assays showed that the more cytotoxic particles were the CuO NPs, which, after 24 h of exposure at concentration of 20 µg/mL, induced a cell mortality over 80%. A comparable toxic effect has been reported in previous studies, in which CuO NPs resulted as more cytotoxic compared to other MeO NPs and carbon nanotubes [39]. Furthermore, a study on A549 cells exposed to the same CuO NPs showed that CuO resulted as cytotoxic at lower concentrations of exposure and that the effects were due to the modality of cell–particle interactions [19].

As CuO NPs, the DEP + CuO mixture induced cytotoxicity on A549 cells in a time- and concentration-dependent manner, even though cytotoxicity in the mixture was lower, probably due to the interaction between DEP and CuO NPs. The cytotoxicity measured by MTT for ZnO NPs and the DEP mixture showed a kind of synergic effect between DEP and ZnO NPs. Indeed, the DEP + ZnO induced an enhanced response with respect to the single particle at the dose 20 µg/mL after 24 h of exposure (Figure 2). A similar trend was found also comparing CFE data from 24 h of exposure and 72 h and this effect was probably due to a change in dynamic toxicity and uptake of NPs. With the CFE assay, this recovery is also evident after treatment with ZnO NPs alone after 72 h (Figure 3).

Nevertheless, it is noticeable that the presence of both NPs in the two different mixtures with DEP enhanced the toxicity of the environmental UFP. DEP did not affect cytotoxicity, but in co-exposure with MeO NPs, a decreased cell viability was observed at both 24 h and 72 h. The augmented biological effects of DEP + CuO, compared to DEP alone, are supported by the work of Guo et al., in which the authors showed a synergistic effect on protein oxidation after the co-exposure to the elemental carbon of PM (carbon black, CB) and Fe_2_O_3_ NPs on lung epithelial cells [40]. In the cited work, the authors proposed that when transition metal oxide NPs, such as Fe_2_O_3_ NPs, but also others, and CB are simultaneously internalized, the CB particles may reduce the bioavailable Fe^3+^ ions within the cells and that lysosomal acidification plays a crucial role in the mechanism. We suggest that this process could be similar for DEP + CuO co-exposure. Nevertheless, Guo and co-workers also suggested that synergistic effects of Fe_2_O_3_ NPs and CB are due to the reduction of Fe^3+^ ions by the carbon black particles attributed to the reactive functional groups on the carbon surface. However, in our work, even if the oxidative potential was not investigated, CuO NPs alone were more toxic compared to the DEP + CuO mixture and that could probably be due to the more bioavailability of extracellular Cu^2+^ ions and to intracellular Cu^2+^ ions derived from lysosomal acidification after CuO NPs uptake (Trojan horse mechanism). Furthermore, it is important to mention that DEPs have different physicochemical properties from CB particles and that the PAHs and metals adsorbed on the carbonaceous core of particles may interact differently with MeO with respect to elemental carbon (CB). Previous works have indeed confirmed the possible interactions of MeO NPs with PAHs, such as B(a)P [35], or with organometallic cation [41].

Furthermore, our data suggest that Cu^2+^ ions release is reduced by the presence of DEP with a corresponding reduction of cytotoxicity of the mixture (27% after 24 h) compared with the single NPs of CuO (43% after 24 h), probably due to an interaction between particles that affects the superficial charge of CuO and the ions release. Although other studies have demonstrated that CuO cytotoxicity depends mostly on its intracellular solubility [42], it has also been reported that extracellular dissolution of Cu^2+^ ions from CuO NPs could contribute to NPs cytotoxicity on A549 cells and this effect depends on NPs size and modes of entry of NPs [43]. Regarding ZnO NPs, their cellular toxicity could be explained by the Zn^2+^ ions released from ZnO NPs in the cellular medium solution [44] and that Zn^2+^ ions also induce inflammatory responses [45], in accordance with our results.

The high percentages of Zn^2+^ ions released by the ZnO NPs and DEP + ZnO mixture, compared to CuO NPs alone and in mixture, support the hypothesis that ions dissolution contributes to ZnO toxicity, as previously demonstrated [46]. The slightly different response towards the DEP + ZnO mixture with respect to the individual NPs of ZnO could be attributed to the possible different mechanisms of interaction and endocytosis of DEP + ZnO NPs aggregates, compared to the single ZnO NPs (Figure 6 and Figure S7).

These data about zinc ions’ contribution to cellular toxicity, augmented by the simultaneous exposure with carbonaceous particles, are in accordance with the work of Wilson and colleagues, in which co-exposure to CB and ZnCl_2_ induced a significant release of TNF-a compared to ZnCl_2_ alone [47]. Furthermore, previous works on PM with different content of metals, in particular zinc, showed that PM enriched with Zn resulted as more toxic compared to other samples [48], and that the depletion of zinc ions by chelating agents determined a reduction of the cytotoxic effects [49]. A change in the toxic kinetics of ZnO NPs in mixture with DEP could explain the recovery of viability after the cytotoxicity peak at 24 h of exposure. We suppose that, after 24 h, the survived fraction of cells is able to make cellular replication and for this reason, an increased cell viability was observed. Moreover, contrary to CuO- and DEP + CuO-exposed ones, the DEP + ZnO-exposed cells did not show severe ultrastructural lesions, which may support the capability of them to survive the Zn-mediated insult, and even the elimination of the internalized particles through exocytosis may not be excluded. Nevertheless, these aspects about toxicity kinetics require further investigation.

The inflammatory response was indeed promoted by ZnO NPs and the respective mixture at the highest dose when toxicity occurs. On the contrary, CuO NPs induced IL-8 release in the lung epithelial cells already at sub-toxic concentrations (10 μg/mL), as previously observed by Mantecca et al. [50]. The co-exposure of CuO with DEP resulted in a significant decrease of IL-8 release compared to CuO NPs alone, and DEP themselves did not promote the release of this protein. These data evidenced once again the interference between these two different particles in inducing the biological responses. A reduction of IL-8 after winter Milan PM_2.5_, which is the major source of combustion particles derived from diesel emissions, has already been reported in a previous work [51], in which the authors suggested that the observed effect was accompanied by altered organization of the actin filaments, that actively participate in the motility of secretory granules [52]. Other pro-inflammatory cytokines are very useful markers for understanding the inflammatory response related to NPs and UFP exposure. In our work, the release of TNF-α, after 24 h of exposure to all treatments, was also investigated, but in our model, the release of this protein was very low and we did not notice remarkable variation in cells exposed to the NPs with respect to the control (data not shown).

Our data on actin cytoskeleton alteration underlined that all the treatments induced an alteration of cells regular morphology and a more elongated morphology of cytoskeleton was evident compared to the typical shape of unexposed cells. Exposure to CuO NPs resulted in an accentuated morphological alteration, disorganization of the cytoskeleton and shrinkage of cells, as evidence of cell death. Cells treated with DEP alone and in mixture with CuO NPs showed more presence of filipodia, while uropodia and actin bundles were visible after exposure to ZnO NPs, alone and in mixture, and to CuO NPs. These data confirm that the exposure to engineered NPs could cause cellular cytoskeletal disturbance [53], with consequences in the alteration in proteins involved in cell migration [54].

In the presence of DEP, enhanced plasma membrane ruffling and presence of stress fibres were observed, in accordance with previous data on bronchial cells exposed to fine PM [55].

Data from the TEM analysis confirmed that all the tested particles are internalized by lung cells. ZnO NPs are visible in cellular vesicles after the invagination of the cellular plasma membrane. The presence of multilamellar bodies enlighten the hypothesis that autophagy is one of the key events involved on the response to MeO NPs, and it may act as a survival mechanism by targeting harmful components to the lysosomes for degradation, as already supported by different in vitro works [56]. However, this aspect needs further investigations. As expected, the presence of CuO NPs in lysosomes after 24 h of exposure has confirmed data from the literature [19,57]. Mitochondria were also compromised by this treatment. Interestingly, agglomerates of DEP + CuO NPs are visible in phagosome, suggesting a different kinetics of this mixture compared to single CuO NPs. As a matter of fact, at a prolonged time of exposure, 48 h for MTT and 72 h for CFE assay, DEP + CuO mixtures were more toxic compared to at the 24 h time point.

These results showed that MeO NPs in mixture with airborne UFP could differently affect lung cell toxicity, evidencing the importance to test the possible synergistic or antagonistic effects of different environmental particles. Furthermore, CuO NPs resulted as the more cytotoxic NPs in this study, also able to induce cytotoxicity, inflammatory response, cytostatic effect and cellular morphological changes.

It is likely that the interaction of DEP with ZnO and CuO NPs may interact and change the surface reactivity between environmental UFP and engineered NPs. Furthermore, this interaction is influenced by the kind of NPs. DEP enriched in Zn^2+^ are more toxic than DEP and ZnO NPs alone, while CuO NPs could be adsorbed on DEP surface, reducing the bioavailability of Cu^2+^ with consequent reduced toxic effects compared to single CuO NPs. However, the co-exposure to MeO NPs and DEP highly increases the toxicity of carbon-based UFP.

Recently, the Air Liquid Interface (ALI) exposure systems received considerable attention as an alternative method to the submerged system in studying the biological effects of engineered NPs [58,59] and DEP [60,61]. However, the widespread adoption of ALI systems is still limited by the minimal efficiency of particles deposition and by the complex strategy needed to characterize the composition of the particles delivered to the cells. In the future, ALI exposure to NPs and UFP would be the preferable reference standard, since it more closely mimics a real exposure scenario. The cellular responsiveness to NPs and UFP at the aerosol/airborne phase could vary a lot with respect to submerged conditions, as previously demonstrated by Lenz et al. [58].

We suppose that, at the ALI, A549 cells would have different responses, especially towards DEP, as evidenced by previous authors [61,62]. Biological outcomes would be influenced by different bioavailability to O_2_ and by a different interaction with NPs in the absence of a lining fluid (e.g., reduction of NPs agglomeration and different NPs uptake). Moreover, since free ions release from NPs will be avoid or reduced at ALI exposure, inflammatory response and oxidative stress could also be modulated. Further experimental evidences on NPs and UFP mixture toxicity under ALI conditions should be generated to address such speculations.

## 5. Conclusions

In the light of providing experimental results more strictly reflecting real-life environmental conditions of exposure to outdoor and indoor NPs, the possible interaction of different hazardous airborne particles, and the final toxicity deriving from the mixture effects, should be considered. Different modes of action (MoA) have been proposed for the combined toxicity of DEP and MeO (i.e., ZnO and CuO) NPs. DEP usually exerts its toxic effects thanks to the compounds adsorbed onto the carbonaceous core, such as PAHs and metals, which trigger oxidative stress, inflammation, DNA damage and cytotoxicity. Similar mechanisms are also somehow evoked by MeO NPs, by direct internalization of NPs in cells or ions dissolution. Our results are in accordance with previous studies, in which the different mechanisms of CuO and ZnO NPs and related ions have been demonstrated. Cu is a transition metal oxide that could generate ROS through the Fenton reaction, and the adverse cellular outcomes may even be worsened by the Trojan horse mechanism exerted by the NP forms. The extra- and intra-cellular release of Cu ions from the surface of the less soluble CuO NPs might have been limited by a sort of passivation effect exerted by DEPs, which can justify the lower toxicity of DEP + CuO. The ZnO NPs trigger cytotoxicity mainly thanks to the massive release of zinc ions, which seemed to be almost unaffected by the presence of DEP. The enhanced toxicity observed for the DEP + ZnO mixture was largely attributable to an increased NPs uptake by the lung epithelial cells.

We have previously demonstrated that the variable content of PAHs and metals in different DEPs is responsible for different biological responses in human bronchial cells [24]. Since real environmental DEPs could have different chemical compositions, due to different driving cycles (e.g., on urban centres, highways etc.), combustion processes, and other external sources, it would be extremely interesting to analyse the effect of sampled DEP, enriched with metals coming from NPs-added fuels or NMs manufacturing sites.

Furthermore, it would be suitable to investigate if, by changing the physicochemical properties of NMs (e.g., size, chemical composition, surface properties, coating, and crystallinity), the co-exposure with environmental UFP may modulate the biological responses. This aspect would be very useful in the design of safer NMs and should be incorporated in the nano-risk assessment frameworks.

## Figures and Tables

**Table 1:** Dynamic light scattering (DLS) analyses of CuO and ZnO NPs and mixtures. Z-average and PdI (polidispersity index) of particles suspended in Milli-Q water and in culture medium, as well as ζ-potential in water, are presented. For the analyses, CuO and ZnO NPs at the concentration of 20 µg/mL were used, while for the mixtures, a suspension of 100 µg/mL of DEP and 20 µg/mL of NPs were prepared. For the analyses of DEP, the concentration of 100 µg/mL was used.

	ZnO	DEP + ZnO	CuO	DEP + CuO	DEP (2975)
Milli-Q Z-average ± SE (nm)	275.7 ± 9	179.7 ± 2	208.43 ± 2	217.07 ± 3	263.03 ± 3
PdI	0.361	0.352	0.209	0.223	0.29
Opti-MEM 1% FBS Z-average ± SE (nm)	314.38 ± 204	207.37 ± 7	464.67 ± 2	275.7 ± 3	320.8 ± 6
PdI	0.63	0.52	0.35	0.22	0.21
Milli-Q Z-potential ± SE (mV)	25 ± 0.13	−19 ± 0.21	12 ± 0.6	−18 ± 0.09	−35 ± 0.52

**Table 2:** Inductively coupled plasma-optic emission spectroscopy (ICP-OES) analysis of metal dissolution from CuO and ZnO NPs and mixtures. The release of ions was evaluated after 3 and 24 h of incubation of NPs (20 µg/mL) and mixtures with DEP (100 µg/mL of DEP and 20 µg/mL of NPs) in cell culture medium. Data were expressed as concentration in ppm ± SE. In the table, percentage of dissolute ions after 3 and 24 h in medium, calculated on the base of ICP-OES analysis, is presented. * nd = not detected.

	ZnO	DEP + ZnO	CuO	DEP + CuO
3 h ppm Zn	9.36 ± 1.1	8.3 ± 0.3	0.1 ± 0.0	0.1 ± 0.0
3 h % Zn	51.2%	51.5%	0%	0%
24 h ppm Zn	11.1 ± 0.1	10.0 ± 0.1	nd	nd
24 h % Zn	69.4%	62.5%	43.3%	26.7%
3 h ppm Cu	nd	nd	2.4 ± 0.1	2.4 ± 0.1
3 h % Cu	0%	0%	14.7%	14.5%
24 h ppm Cu	nd	nd	7.1 ± 0.2	4.4 ± 0.2
24 h % Cu	0%	0%	43.3%	26.7%

## Supplementary Materials

The following are available online at https://www.mdpi.com/2079-4991/9/9/1302/s1, Table S1: Scheme describing CFE assay set-up. Figure S1: Cell viability of A549 after exposure to DEP. Figure S2: Colony forming efficiency (CFE) assay. Figure S3: Analysis of the mean area of the colony. Figure S4: Inflammatory response. Figure S5: Inflammatory response at 24 h. Figure S6: Inflammatory response at 3 h. Figure S7: TEM images of A549 cells exposed to ZnO NPs and DEP + ZnO NPs.
