# Supplementary material for: Mixture Effects of Diesel Exhaust and Metal Oxide Nanoparticles in Human Lung A549 Cells
Source: Nanomaterials (Basel). 2019 Sep 11;9(9):1302. doi: 10.3390/nano9091302 (PMC6781047; doi:10.3390/nano9091302)
Supplement: Supplementary file 1 [file nanomaterials-09-01302-s001.pdf]

Supplementary Information

Mixture Effects of Diesel Exhaust and Metal Oxide Nanoparticles in Human Lung A549 Cells

Alessandra Zerboni [1,*], Rossella Bengalli [1], Giulia Baeri [1], Luisa Fiandra [1], Tiziano Catelani [2] and Paride Mantecca [1,*]

[1] POLARIS Research Center, Department of Earth and Environmental Sciences, University of Milano - Bicocca, Piazza della Scienza 1, 20126 Milan, Italy; a.zerboni2@campus.unimib.it; rossella.bengalli@unimib.it; g.baeri@campus.unimib.it; luisa.fiandra@unimib.it; paride.mantecca@unimib.it

[2] Microscopy facility, University of Milano-Bicocca, Piazza della Scienza 3, 20126 Milano, Italy; tiziano.catelani@unimib.it.

* Correspondence: a.zerboni2@campus.unimib.it (A.Z.); paride.mantecca@unimib.it (P.M.); Tel.: +39–02–6448–2916 (P.M.)

Table S1. Scheme describing CFE assay set-up.

DAY 1	DAY 2	DAY 3	DAY 4	DAY 5	DAY 6	DAY 7	DAY 8
Cell seeding	Exposure T = 0 h	T = 24 h		T = 72 h			Fix and stain

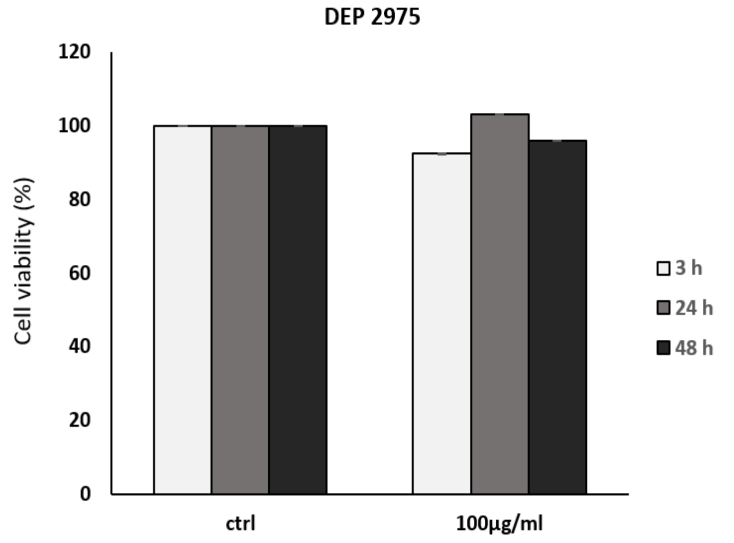

Figure S1. Cell viability of A549 after exposure to DEP. Histograms represent the percentage respect to control cells (100%) of viable cells after the exposure to DEP 100 μg/ ml. Data show the mean ± SE (n = 3).

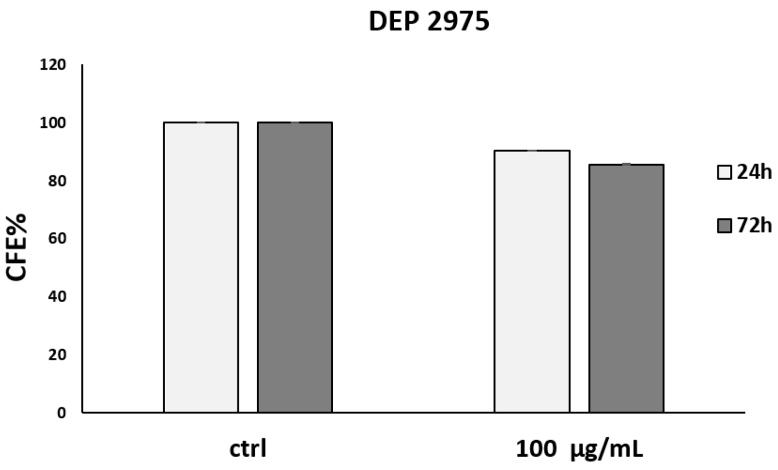

Figure S2. Colony forming efficiency (CFE) assay. Histograms represent the percentage of CFE calculated after the exposure to DEP 100 µg/mL. Data show the mean ± SE (n = 3).

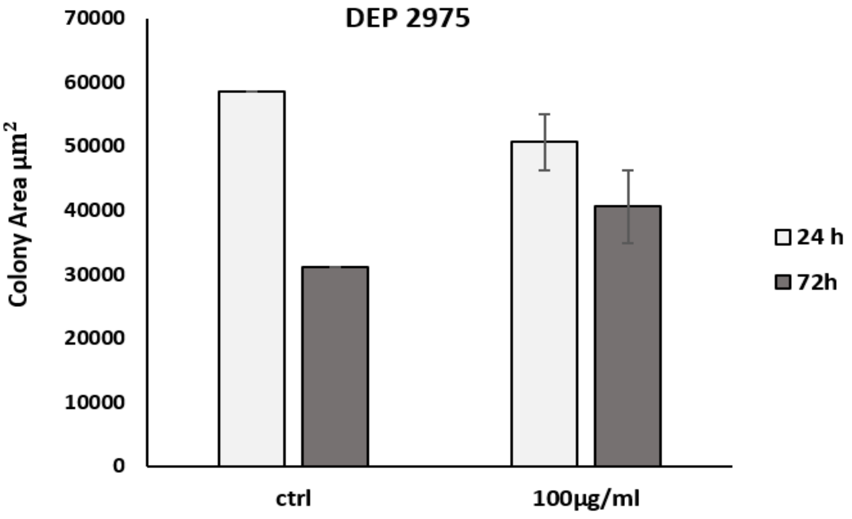

Figure S3. Analysis of the mean area of the colony. Mean of 10 counts, ± SE.

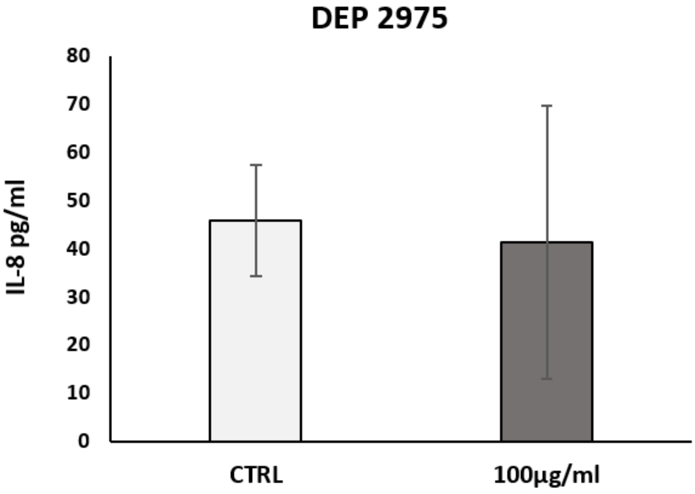

Figure S4. Inflammatory response. The release of the pro-inflammatory cytokine IL-8 was evaluated in A549 supernatants after the exposure for 24 h to DEP (100 µg/mL). Data are presented as pg/mL and the histograms represent the mean ± SE of at least three independent experiments.

A

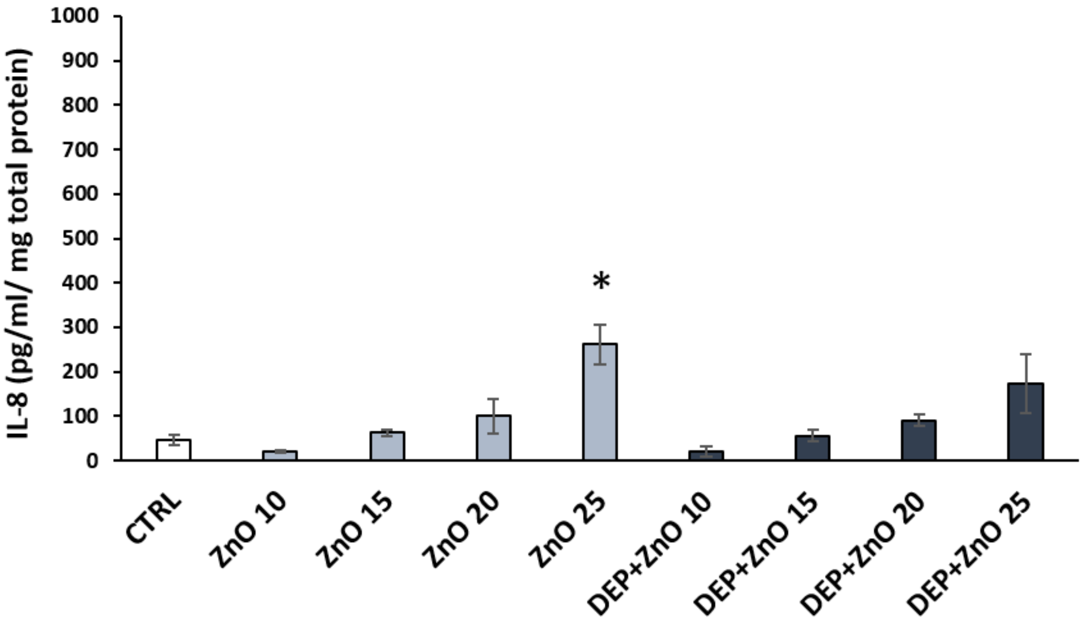

B

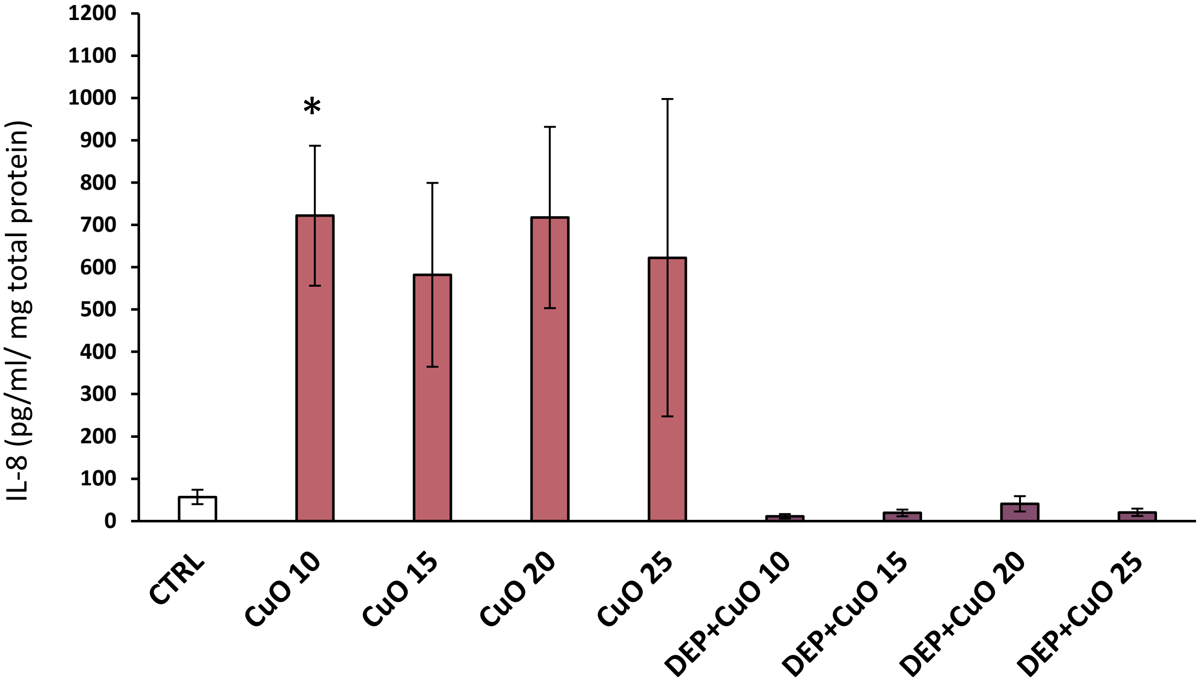

Figure S5. Inflammatory response at 24 h. The release of the pro-inflammatory cytokine IL-8 was evaluated in A549 supernatants after the exposure for 24 h to: A) ZnO (10, 15, 20 and 25 µg/mL) and ZnO+DEP (100 µg/mL); B) CuO (10, 15, 20 and 25 µg/mL) and CuO+DEP (100 µg/mL). Data were normalized to the total amount of protein (mg) in each sample measured by BCA (Bicinchoninic Acid) Protein Assay (Sigma Aldrich), which is relate to the number of cells. Data are presented as pg/mL/mg total protein and the histograms represent the mean ± SE of at least three independent experiments.

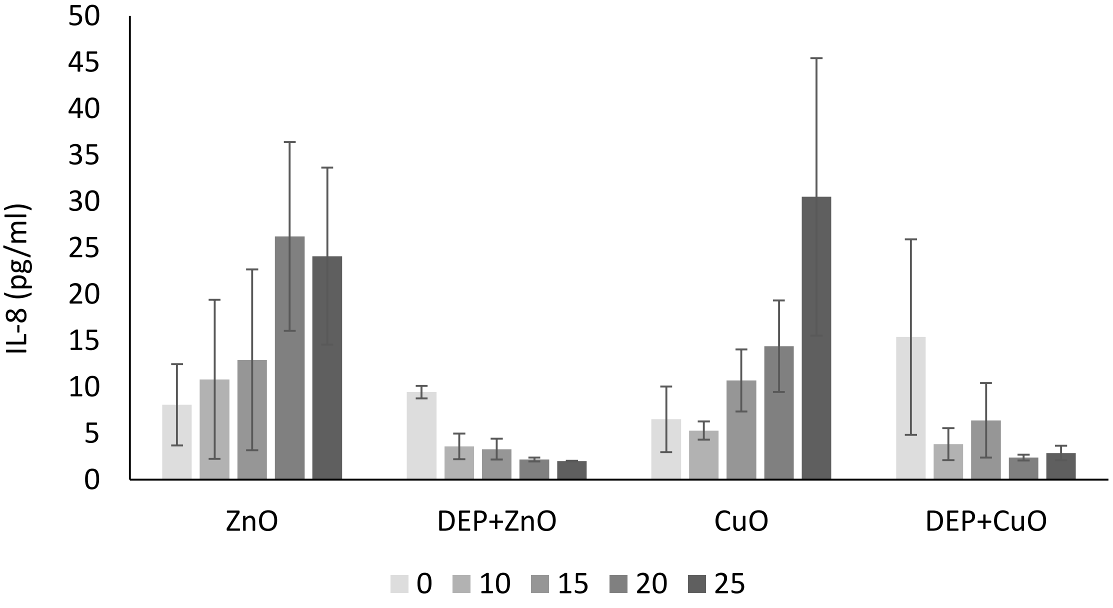

Figure S6. Inflammatory response at 3 h. The release of the pro-inflammatory cytokine IL-8 was evaluated in A549 supernatants after the exposure for 3h to 0, 10, 15, 20, 25 µg/mL of ZnO and CuO NPs alone and mixed with DEP (100 µg/mL). Data are presented as pg/mL and the histograms represent the mean ± SE of at least three independent experiments.

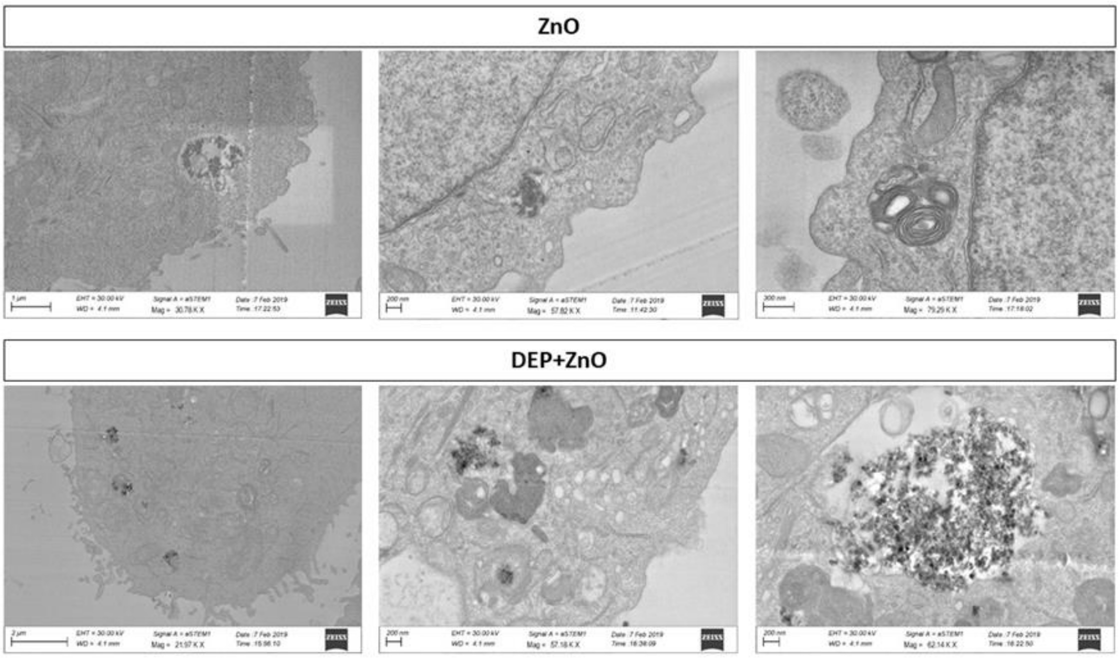

Figure S7. TEM images of A549 cells exposed to ZnO NPs (upper panel) and DEP + ZnO NPs (lower panel). Cells exposed to ZnO showed low NP uptake and only few multivesicular bodies with debris and particulate inclusions were observable in the cytoplasm; cells exposed to DEP + ZnO showed several and large vesicles containing cellular debris together with electron dense NPs. Cells were exposed for 24h to ZnO NPs at 20 μg/mL and DEP at 100 μg/mL.
